# Inflammatory Cytokine-Producing Cells and Inflammation Markers in the Synovium of Osteoarthritis Patients Evidenced in Human Herpesvirus 7 Infection

**DOI:** 10.3390/ijms21176004

**Published:** 2020-08-20

**Authors:** Valerija Groma, Mihails Tarasovs, Sandra Skuja, Sofija Semenistaja, Zaiga Nora-Krukle, Simons Svirskis, Modra Murovska

**Affiliations:** 1 Joint Laboratory of Electron Microscopy, Institute of Anatomy and Anthropology, Riga Stradins University, Kronvalda blvd 9, LV-1010 Riga, Latvia; mihails.tarasovs@rsu.lv (M.T.); Sandra.skuja@rsu.lv (S.S.); sofijasem@inbox.lv (S.S.); 2 Department of Internal Diseases, Riga Stradins University, Hipokrata str. 2, LV-1038 Riga, Latvia; 3 Institute of Microbiology and Virology, Riga Stradins University, Ratsupites str. 5, LV-1067 Riga, Latvia; Zaiga.nora@rsu.lv (Z.N.-K.); ssvirskis@latnet.lv (S.S.); modra.murovska@rsu.lv (M.M.)

**Keywords:** osteoarthritis, synovium, cytokines, HHV-7, PCR, ELISA, immunohistochemistry

## Abstract

A direct association between joint inflammation and the progression of osteoarthritis (OA) has been proposed, and synovitis is considered a powerful driver of the disease. Among infections implicated in the development of joint disease, human herpesvirus 7 (HHV-7) infection remains poorly characterized. Therefore, we assessed synovitis in OA patients; determined the occurrence and distribution of the HHV-7 antigen within the synovial membrane of OA-affected subjects; and correlated plasma levels of the pro-inflammatory cytokines tumor necrosis factor (TNF), interleukin-6 (IL-6), and TNF expressed locally within lesioned synovial tissues with HHV-7 observations, suggesting differences in persistent latent and active infection. Synovial HHV-7, CD4, CD68, and TNF antigens were detected immunohistochemically. The plasma levels of TNF and IL-6 were measured by an enzyme-linked immunosorbent assay. Our findings confirm the presence of persistent HHV-7 infection in 81.5% and reactivation in 20.5% of patients. In 35.2% of patients, virus-specific DNA was extracted from synovial membrane tissue samples. We evidenced the absence of histopathologically detectable synovitis and low-grade changes in the majority of OA patients enrolled in the study, in both HHV-7 PCR+ and HHV-7 PCR‒ groups. The number of synovial CD4-positive cells in the HHV-7 polymerase chain reaction (PCR)+ group was significantly higher than that in the HHV-7 PCR‒ group. CD4- and CD68-positive cells were differently distributed in both HHV-7 PCR+ and HHV-7 PCR‒ groups, as well as in latent and active HHV-7 infection. The number of TNF+ and HHV-7+ lymphocytes, as well as HHV-7+ vascular endothelial cells, was strongly correlated. Vascular endothelial cells, especially in the case of infection reactivation, appeared vulnerable. The balance between virus latency and reactivation is a long-term relationship between the host and infectious agent, and the immune system appears to be involved in displaying overreaction when a shift in the established equilibrium develops.

## 1. Introduction

Joint diseases are recognized as common, widespread disabling pathologies all over the globe [1]. Among chronic rheumatic diseases having a substantial impact on population health, osteoarthritis (OA) is the one destined to increase and become the most prevalent [2]. It is estimated that among persons with OA, about 80% have some degree of movement limitation and 25% are unable to perform daily activities [3]. Previous studies have accentuated the role of OA as the major cause of hip and knee replacement surgeries [4]. Osteoarthritis has long been viewed as a degenerative disease of cartilage, but accumulating evidence indicates that inflammation has a crucial role in its pathogenesis [5,6]. A direct association between joint inflammation and the progression of OA has been proposed [7,8], and synovitis has been considered a powerful driver of the OA process [9].

The synovial membrane comprises a tissue enclosing the synovial cavity around the opposing surfaces of articular cartilage. It contains a superficial layer, called the intima, composed of two types of synoviocytes—macrophages or type A cells and fibroblast-like or type B cells—and underneath is layer of subintima which houses blood vessels and nerves. Synoviocytes manufacture, secrete, absorb, and adjust the contents of the joint cavity by producing and remodeling the extracellular matrix molecules (ECMs), both collagenous and ground substance/adhesion molecules, thus controlling local homeostasis [10,11]. Intimal cells are essential producers of cytokines, including the pro-inflammatory factors tumor necrosis factor (TNF), interleukin-1β (IL-1β), and interleukin-6 (IL-6) [12,13,14]. Cytokines diffusing through the synovial fluid into the articular cartilage may further activate chondrocytes and synoviocytes, thus sustaining inflammation [15].

Arthritogenic viruses implicated in the development of joint pathologies [16], including those manifesting with synovial damage, have been explored [16,17,18,19]. A viral etiology is evident for approximately 1% of all cases of acute arthritis [20]. Human herpesviruses are ubiquitous pathogens establishing a persistent infection in the host for life, but their contribution to articular damage and the etiopathogenesis of OA remains obscure [21,22,23,24].

The results of several studies have confirmed the presence of human herpesvirus 6 (HHV-6) and 7 (HHV-7) DNA and viral antigens by polymerase chain reaction (PCR) techniques, in situ hybridization, immunohistochemistry, and electron microscopy in blood plasma, peripheral blood mononuclear cells, the brain, and skin [25,26,27,28,29,30]. Furthermore, our earlier study reported on the presence of human HHV-6 and HHV-7 infection markers in synovial fluid and synovial tissues of rheumatoid arthritis (RA)-affected patients [31]. HHV-6A, HHV-6B, and HHV-7 are genetically related to human cytomegalovirus (HCMV) constituting the β-herpesvirus subfamily [22,32]. There is evidence suggesting that Epstein–Barr virus (EBV) and HCMV infection contribute to the pathogenesis of RA [18]. Furthermore, the presence of DNA from varicella zoster (VZV), herpes simplex virus (HSV), EBV, and HHV-6 has been confirmed in the synovial fluid and peripheral blood mononuclear cells of patients with RA, OA, and axial spondyloarthritis. In RA and spondyloarthritis, the authors found that the PCR results were concordant with the inflammatory activity of the disease [33,34]. The frequency and extent of synovial inflammation in OA linked to the assessment of inflammatory cytokine-producing cells evidenced in the presence of HHV-7 infection has not yet been elucidated, including the latency and reactivation conditions.

## 2. Results

### 2.1. Nested Polymerase Chain Reaction

**Figure 1:**
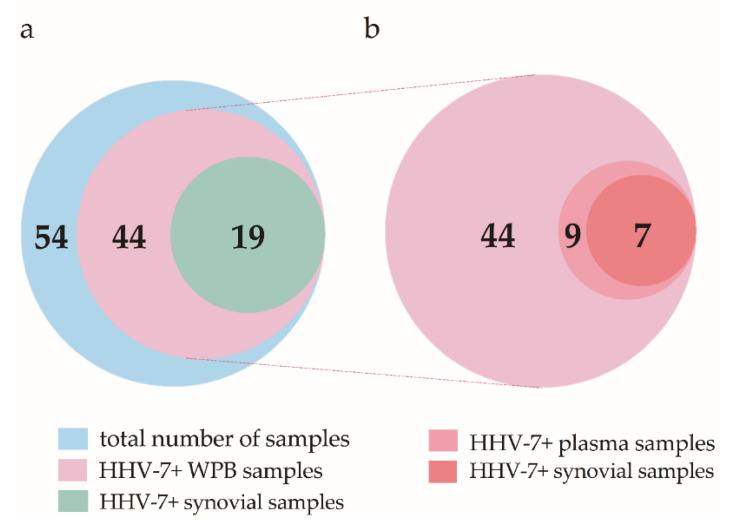
Distribution of patients enrolled in the study according to nested PCR data. (**a**) Venn diagram circles are scaled according to the number of samples and depict the total number, the number of human herpesvirus 7 (HHV-7)-positive whole peripheral blood (WPB), and the synovial membrane tissue samples. (**b**) Extract from the HHV-7 PCR+ group depicts the number of positive samples detected in the case of persistent and active viral infection (blood plasma and synovial membrane samples).

Qualitative nested PCR (nPCR) testing was performed on 54 patients. The presence of persistent HHV-7 infection (the presence of the HHV-7 genomic sequence in DNA extracted from whole peripheral blood (WPB)) was detected in 44/54 (81.5%) OA patients. Out of 44 OA patients, the HHV-7 sequence in WPB DNA samples was detected in 27 females and 17 males. In 19/54 (35.2%) patients, virus-specific DNA was also present in DNA extracted from synovial membrane tissue samples (Figure 1a). All samples from patients with HHV-7 genomic sequences in whole blood DNA were analyzed for viral infection reactivation (viral genomic sequences in cell-free blood plasma DNA). HHV-7 reactivation was found in 9/44 (20.5%) patients. Interestingly, in seven out of nine patients with an active viral infection, the HHV-7 specific genomic sequence was also found in both WPB and synovial membrane DNA (Figure 1b).

### 2.2. Plasma Levels of TNF and IL-6

**Figure 2:**
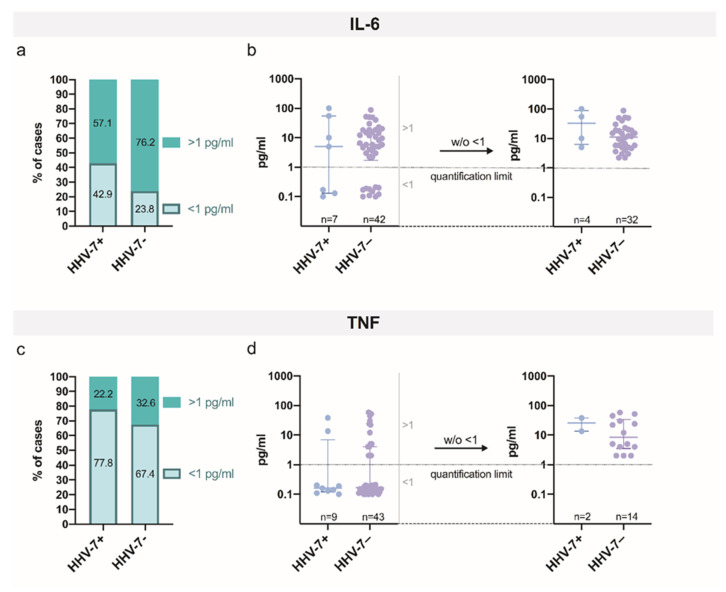
Assessment of plasma levels of pro-inflammatory cytokines—tumor necrosis factor (TNF) and interleukin-6 (IL-6)—in osteoarthritis (OA) patients in both study groups, HHV-7 PCR+ and HHV-7 PCR−. Plots depict the distributions of IL-6 (**a**) and TNF (**c**) plasma levels demonstrated in OA patients in both study groups—HHV-7 PCR+ and HHV-7 PCR−. (**b**,**d**) Dot plots represent the quantified data for IL-6 (**b**) and TNF (**d**) plasma levels. (**b**) Each dot represents a single data point; blue dots represent IL-6 plasma levels assessed in the HHV-7 PCR+ group, and violet dots represent IL-6 plasma levels assessed in the HHV-7 PCR− group. (**d**) Each dot represents a single data point; blue dots represent TNF plasma levels assessed in the HHV-7 PCR+ group, and violet dots represent TNF plasma levels assessed in the HHV-7 PCR− group. The quantification limit (QL) for the assay of cytokine assessment was set as 1 pg/mL. Cytokine levels below QL (<1) were uniformly set as random values around QL/10. The right part of graph (**b**) and graph (**d**) represent data excluding values below the detection (quantification) level (w/o < 1), i.e., data without values of less than 1 (w/o < 1). W/o—without (write-off).

The plasma levels of both pro-inflammatory cytokines greatly varied from 0 to 58 pg/mL and from 0 to 100 pg/mL for TNF and IL-6, respectively. No significant difference in the plasma levels for TNF or IL-6 was determined when the HHV-7 PCR+ and HHV-7 PCR− groups were compared. Plots representing the distributions of IL-6 and TNF cytokine plasma levels found in OA patients of both study groups, consisting of HHV-7 PCR+ and HHV-7 PCR−, can be seen in Figure 2.

### 2.3. Assessment of Synovitis Applying the Krenn Scoring System

**Figure 3:**
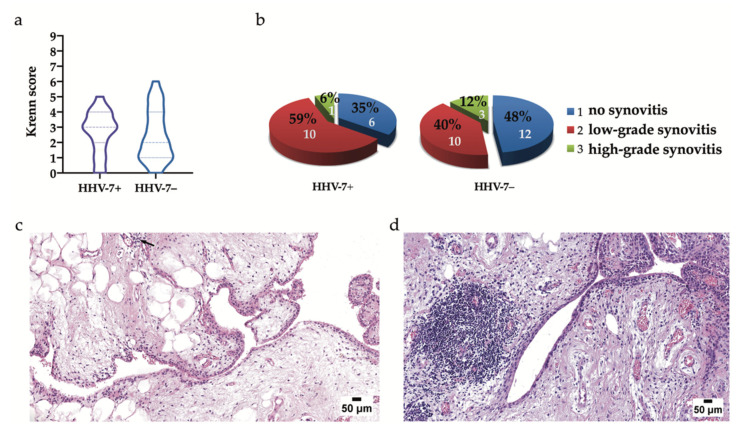
The presence of synovitis assessed by Krenn scores (**a**), statistically (**b**), and morphologically (**c**). (**a**) Violin plots depict the differences in median values demonstrated in the histopathological assessment of Krenn scores from the study groups. Synovitis revealed in the tissue samples of HHV-7 PCR+ OA patients presented with a median value of 3 (IQR 2–4), whereas HHV-7 PCR− OA patients presented with a median value of 2 (IQR 1–4). There was no significant difference found in the synovitis scores estimated for the HHV-7 PCR+ and HHV-7 PCR− groups (*p* = 0.483). Simultaneously, Krenn scores tended to be higher in the HHV-7 PCR+ group when compared to the HHV-7 PCR− group. (**b**) Frequencies of the absence of synovitis and the presence of low- and high-grade synovitis detected during histopathological assessment of the synovial membrane tissue samples from the study groups. The estimation confirms that OA patients commonly present without histopathologically detectable synovitis or demonstrate low-grade synovial inflammation. (**c**) A representative image depicting low-grade synovitis in OA. The synovial lining layer is slightly thickened, and the stromal density is slightly increased; few perivascular lymphocytes are evident (arrow). Hematoxylin and eosin staining. (**d**) A low-power image demonstrating high-grade synovitis in OA. The synovial lining layer is moderately thickened, and some lymphocytes are evident; stroma reveals moderate activation, whereas perivascular inflammatory cells and lymphatic follicle characterize the inflammatory component. Hematoxylin and eosin staining. Scale bars: 50 μm.

Forty-eight OA patients out of a cohort of 54 presented with materials sufficient for further analyses and were stratified into two groups: Nineteen HHV-7 PCR+ subjects (39.6%) and 29 HHV-7 PCR− subjects (60.4%). Seventeen HHV-7 PCR+ and 25 HHV-7 PCR− subjects presented with tissues suitable for assessing synovitis. HHV-7 PCR+ OA subjects presented with a median synovitis score of 3 (IQR 2–4), whereas HHV-7 PCR− OA subjects had a median synovitis score of 2 (IQR 1–4). The trend towards higher Krenn scores in the HHV-7 PCR+ group when compared to the HHV-7 PCR− group was confirmed (Figure 3a). There was no significant difference found in synovitis scores estimated for the HHV-7 PCR+ and HHV-7 PCR− groups (*p* = 0.483). Six (35%), 10 (59%), and 1 (6%) and 12 (48%), 10 (40%), and 3 (12%) OA patients presented without histopathologically detectable synovitis, low-grade synovitis, and high-grade synovitis in the HHV-7 PCR+ and HHV-7 PCR− study groups, respectively (Figure 3b).

### 2.4. Histopathology and Immunohistochemical Detection of Antigens within the Synovial Membrane

To better explore the extent of synovitis and the contribution of cells to the development of inflammation, we performed a microscopical analysis of the synovial membrane tissue samples. In the first set of histopathological examinations, we assessed the synovial morphology in HHV-7 PCR+ and HHV-7 PCR− groups when inflammation was not confirmed microscopically. Histopathologically, the lining cells formed one layer, the synovial stroma revealed normal cellularity, and no inflammatory infiltrates were present. In contrast, the synovial lesions consistent with low-grade synovitis demonstrated an increase in thickness of the lining layer and stromal cellularity, and the presence of a few, mostly perivascular lymphocytes or/and plasma cells (Figure 3c). Comparatively, high-grade synovitis was distinguished by the presence of a greatly thickened lining; the appearance of ulceration and multinucleated giant cells; greatly increased stromal cellularity; and, finally, the presence of numerous lymphocytes and plasma cells, often forming follicle-like aggregates (Figure 3d).

**Figure 4:**
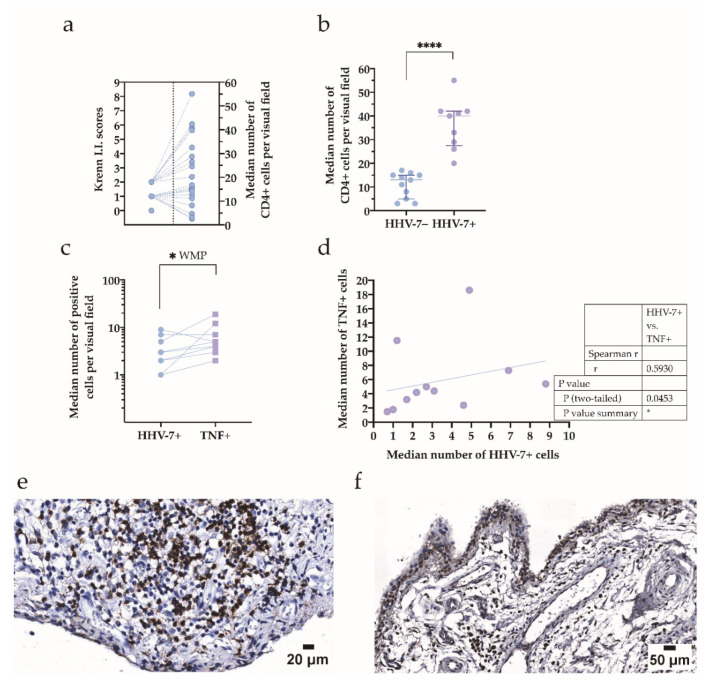
Assessment of synovial CD4-positive lymphocytes and TNF-positive cells in both study groups—HHV-7 PCR+ and HHV-7 PCR−—and HHV-7-positive cells in the HHV-7 PCR+ group. (**a**) The absence of synovitis graded as 0–1, and the presence of a small number of synovial CD4-positive lymphocytes found in the samples of both study groups—HHV-7 PCR+ and HHV-7 PCR−—consistent with low-grade synovitis (grade “2”). Grade “2” synovitis presented with a higher number of CD4-positive lymphocytes when compared to the lower grades. I.I. Krenn—inflammatory infiltration as a substantial part of the Krenn score (inflammatory infiltration, cellular hyperplasia of the lining layer, and cellular density of the sublining layer summed up to provide the Krenn score). Each dot represents a single data point. (**b**) The median number of CD4-positive cells per visual field in the synovial samples obtained from the HHV-7 PCR+ group is significantly higher than that in the HHV-7 PCR− group. Asterisks represent the significance level (**** *p* < 0.0001). (**c**) Median numbers assessed for immunohistochemically confirmed that positive cells are plotted for TNF and HHV-7 antigens. WMP—Wilcoxon matched-pairs signed rank test. The asterisk represents the significance level (* *p* < 0.05). Each dot represents a single data point; blue dots represent HHV-7+ cells, and violet squares represent TNF+ cells. (**d**) Correlation of the median number of HHV-7+ and TNF+ cells expressed per visual field and detected in the samples of the HHV-7 PCR+ group; *r* = 0.593, *p* = 0.0453. An increase in the number of HHV-7+ cells reveals the elevation in the number of TNF+ cells. (**e**) CD4 immunohistochemistry. A representative image demonstrating T-lymphocytes decorated by the anti-CD4 antibody and recognized by the presence of brown reaction products in a follicle-like lymphocytic inflammatory infiltrate found in the synovial sample of HHV-7 PCR+ subjects. Scale bar 20 μm. (**f**) Through the use of TNF immunohistochemistry, the synovial lining presents TNF-positive cells interspersed by TNF-negative cells, whereas the sublining demonstrates mostly perivascular positivity observed in a sample of HHV-7 PCR+ patients. Scale bar: 50 μm.

To further explore synovitis, we specified the cellular contributors by the use of immunohistochemistry. The small number of synovial CD4-positive lymphocytes found in the samples of both study groups, consisting of HHV-7 PCR+ and HHV-7 PCR−, was in line with low-grade synovitis (Figure 4a). Even in the absence of severe synovial inflammation, a statistically significant difference between the number of CD4-positive cells in HHV-7 PCR+ and HHV-7 PCR‒ groups was confirmed (Figure 4b).

To better assess the local expression of the pro-inflammatory marker TNF, we compared the numbers of TNF-positive cells in HHV-7 PCR+ and HHV-7 PCR− OA, and found no statistically significant differences between the groups. Similarly, no correlation was established when plasma cytokine levels were compared to the data depicting immunohistochemistry findings. Furthermore, we used the Wilcoxon matched-pairs signed rank test to compute the matched pairs, TNF-positive cell number, and HHV-7-positive cell number, submitting synovial samples of the HHV-7 PCR+ group to the test (Figure 4c). Simultaneously, in the HHV-7 PCR+ group, changes in the TNF-positive and HHV-7-positive cell count had a significant, positive correlation (*r* = 0.593, *p* = 0.0453) when assessed by Spearman’s rank correlation (Figure 4d).

When submitting the synovial samples obtained from both study groups for microscopical analysis, the lymphocytes and plasma cells colonizing the sublining layer demonstrated either diffusely scattered patterns of distribution, or compact and mostly perivascular patterns. Often, the presence of small follicle-like lymphocytic inflammatory infiltrates was confirmed (Figure 4e). Simultaneously, when assessed immunohistochemically, TNF-positive cells were distributed across the synovial lining and sublining and often demonstrated perivascular localization (Figure 4f).

**Figure 5:**
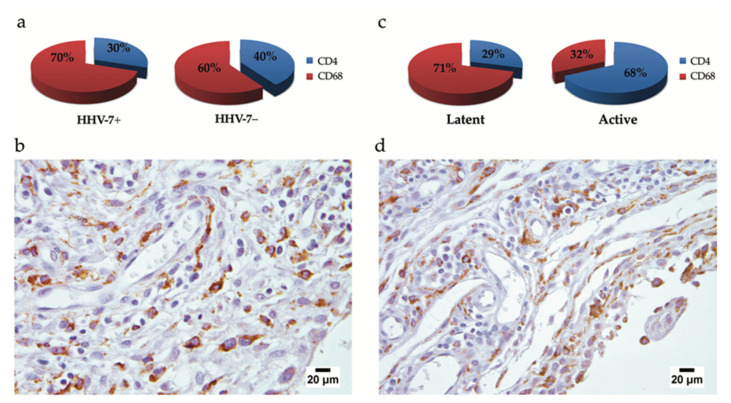
Comparison of the distribution of synovial CD68- and CD4-positive inflammatory cells by the use of statistics (**a**,**c**), and the microscopical assessment of CD68-positive cells (**b**,**d**). (**a**) The distribution of CD68- and CD4-positive cells in both HHV-7 PCR+ and HHV-7 PCR− groups: CD68-positive cells constituted almost two-thirds (70%) of inflammatory cells found in HHV-7 PCR− samples, whereas, for CD68- and CD4-positive cells, 60 and 40%, respectively, were more equally distributed in HHV-7 PCR+. (**c**) CD68-positive cells represented a major part (71%) of inflammatory cells in latent HHV-7 infection, whereas these were opposingly distributed in active HHV-7 infection, with 68% and 32% of CD4- and CD68-positive cells, respectively. (**b**,**d**) CD68 immunohistochemistry; a representative image ((**b**), HHV-7 PCR− sample; (**d**), HHV-7 PCR+ sample) demonstrating CD68-positive cells decorated by the anti-CD68 antibody and developed brown reaction products in the synoviocytes of lining layer and the macrophages of sublining layer. The immunohistochemical decoration reflects the presence of lysosome-specific proteins involved in sorting in the trans-Golgi region, targeting to lysosomes, and fusion with the plasma membrane.

To better recognize and estimate residential cells, synovial macrophages, and their role in the production of pro-inflammatory cytokines, we labeled cells with the anti-CD68 antibody. Furthermore, we compared the presence and number of CD68-positive and CD4-positive cells. The distribution of inflammatory cells bearing CD68 and CD4 labeling varied in both HHV-7 PCR+ and HHV-7 PCR− groups; however, the difference was not statistically significant (Figure 5a). Opposingly, the distribution of synovial CD68-positive cells and CD4-positive cells differed to a greater extent when latent and active HHV-7 infection was referred (Figure 5c). Simultaneously, no correlation was established when CD68 immunohistochemistry data were compared to the results depicting plasma pro-inflammatory cytokine levels (TNF and IL-6). Under the microscope, CD68-positive cells presented in both synovial subdivisions, the lining and sublining layers were diffusely distributed in both HHV-7 PCR− (Figure 5b) and HHV-7 PCR+ (Figure 5d) groups, and more local patterns of distribution were acquired when contributing to follicle-like inflammatory infiltrates.

**Figure 6:**
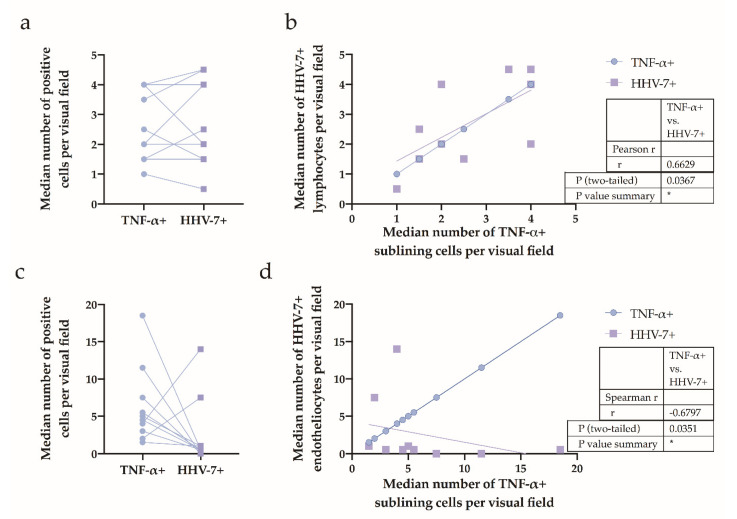
Assessment of synovial HHV-7-positive cells—lymphocytes (panel (**a**) and (**b**)) and vascular endothelial cells (panel (**c**) and (**d**)), and TNF-positive cells in the HHV-7 PCR+ group. (**a**) Median numbers assessed for immunohistochemically confirmed positive cells are plotted for the TNF and HHV-7 antigen. Each dot represents a single data point; blue dots represent TNF+ cells, and violet squares represent HHV-7+ lymphocytes. (**b**) Correlation between the median number of TNF+ cells and HHV-7+ lymphocytes expressed per visual field and detected in the samples of the HHV-7 PCR+ group; *r* = 0.6629, *p* = 0.0367. Each dot represents a single data point; blue dots represent TNF+ cells, and violet squares represent HHV-7+ lymphocytes. Correlations determined by Pearson’s rank correlation test. (**c**) Median numbers assessed for immunohistochemically confirmed positive cells are plotted for the TNF and HHV-7 antigen. Each dot represents a single data point; blue dots represent TNF+ cells, and violet squares represent HHV-7+ endotheliocytes. (**d**) Correlation between the median number of TNF+ cells and HHV-7+ endotheliocytes expressed per visual field and detected in the samples of the HHV-7 PCR+ group; *r* = −0.6797, *p* = 0.0351. Each dot represents a single data point; blue dots represent TNF+ cells, and violet squares represent HHV-7+ endotheliocytes. Correlations were determined by the Spearman’s rank correlation test.

Fourteen of 19 HHV-7 PCR+ OA patients presented with synovium applicable for further immunohistochemical studies and tissue detection of the antigen. Furthermore, HHV-7-positive lymphocytes and macrophages were distinguished by their cytological appearance. To better explore the relationship between synovial cells bearing the HHV-7 antigen and TNF-producing cells, we computed the matched pairs’ TNF-positive cell number and HHV-7-positive cell number, similar to Figure 4c,d but stratified into cellular types (Figure 6a,c). We determined a statistically significant, positive correlation (*r* = 0.6629, *p* = 0.0367) between HHV-7-positive lymphocytes and TNF-positive cells (Figure 6b), whereas a negative correlation (*r* = −0.6797, *p* = 0.0351) was found between HHV-7-positive endotheliocytes and TNF-positive cells (Figure 6d).

**Figure 7:**
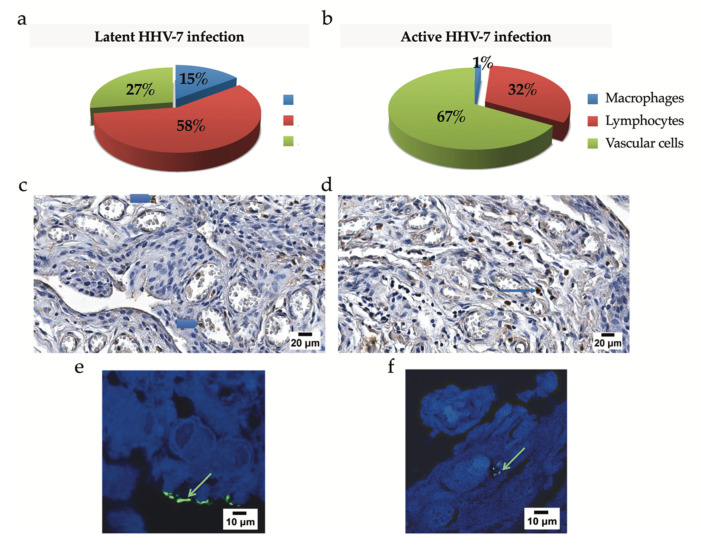
The assessment of the HHV-7 antigen in the synovial membrane. (**a**,**b**) Frequencies of HHV-7-positive lymphocytes, vascular endotheliocytes, and macrophages based on immunohistochemistry data and compared for patients presenting with latent (**a**) and active infection (**b**). When assessed quantitatively, HHV-7-positive lymphocytes constituted a significant cellular fraction affected by the virus in latent HHV-7 infection and reached 58%. In contrast, vascular endotheliocytes forming the innermost layer of vascular beds appeared the most vulnerable in active HHV-7 infection, demonstrating a 67% involvement. There was a significant difference found between the distribution of HHV-7-positive endotheliocytes estimated for latent and active HHV-7 infections (*p* = 0.028). (**c**) Immunohistochemical detection of the HHV-7 antigen in the case of latent HHV-7 infection. Intravascular HHV-7-positive cells recognized by brown coloration (blue arrowheads) localized in the lumen of congested blood vessels. Cellular nuclei counterstained with Mayer’s hematoxylin (blue). Scale bar: 20 μm. (**d**) Numerous perivascular (blue arrow) and vascular endothelial HHV-7-positive cells localized in the sublining layer. Cellular nuclei counterstained with Mayer’s hematoxylin (blue). Scale bar: 20 μm. Detection of the tegument protein pp85 of HHV-7 by immunofluorescence (HHV-7-immunopositive products, green), confocal microscopy; 1% toluidine blue was added to the fluorophore, and it resulted in near infra-red fluorescence in the cellular cytoplasmic compartment. Green arrows indicate the presence of viral protein at the top of synovial macrophages (**e**) and within the cell cytoplasm (**f**). Scale bar: 10 μm.

Finally, HHV-7 immunohistochemistry data were compared for patients presenting with latent and active infection. When assessed quantitatively, HHV-7-positive lymphocytes, endotheliocytes, and macrophages constituted 58, 27, and 15% and 32, 67, and 1% of the cases of latent and active HHV-7 infection, respectively (Figure 7a,b). The immunohistochemical estimation of synovial HHV-7-positive cells applied for the HHV-7 PCR+ group demonstrated that cells labeled with the anti-HHV-7 antibody were localized in the sublining layer, both peri- and intravascularly (Figure 7c,d), and in the lining layer (Figure 7e,f). Moreover, some endothelial cells constituting the internal lining of blood vessels found in the synovial membrane stroma were positively stained with the anti-HHV-7 antibody. Furthermore, evidence of the presence of HHV-7 expression in synovial tissue correlated with nPCR data.

## 3. Discussion

Chronic, low-grade, local inflammation underlining the OA process has been considered as an essential feature of the disease [6,8,9,35]. Furthermore, the action of an inflamed synovium as a trigger of the OA process has been suggested and points at cells recruited in intra-articular changes [36]. Therefore, the assessment of synovial lesions has been encouraged by both research and clinical practice [37,38,39].

Previous studies have explored the molecular mechanisms of the cell entry of betaherpesviruses; some authors have designated CD46 as an entry receptor in the case of HHV-6 infection and demonstrated the presence of it on a major target cell—activated T lymphocytes [40]. Alternatively, other authors have explored CD134, which is a receptor specific for HHV-6B, belonging to the TNF receptor superfamily and expressed on activated CD4-positive T cells [41,42,43].

Human herpesvirus 7, one of the most prevalent viruses in the human population, has also been recognized to target lymphocytes [44]. Furthermore, the activation of infected T lymphocytes leading to the reactivation of HHV-7 has been confirmed [45]. Finally, in HCMV infection, up to 30% of all circulating CD4-positive T lymphocytes become the primary target of the virus in infected elderly individuals. Furthermore, the authors showed that these HCMV-affected T lymphocytes may contribute to significant shifts in the leukocyte composition of peripheral blood and an increase in the number of “effector-memory” T cells [46].

Importantly, the OA synovium has been reported to be a tissue enriched in T cells when compared to the normal synovial membrane [38,39,47,48,49], and the proportion of CD4-positive T cells and the CD4-positive/CD8-positive ratio found in peripheral blood are recognized as being significantly higher in OA patients when compared to healthy controls [7,50]. Other authors have reported on synovial tissue damage induced by CD4-positive T cells and evidenced, at a later stage of the disease, that activated CD4-positive T cells promote lesions and induce macrophage inflammatory protein-1γ expression and subsequent osteoclast formation in OA patients [51]. Given that the demonstrated overall assessment of CD4-positive T cells is in line with synovial morphological findings, this suggests a clear predominance of OA subjects without histopathologically detectable and low-grade inflammation confirmed in both study groups. In this study, the median number of CD4-positive cells per visual field assessed in the synovial samples of the HHV-7 PCR+ group was significantly higher than that in the HHV-7 PCR‒ group (*p* < 0.0001). The role of HHV-7 infection in the development and progression of either synovitis or OA remains largely unknown. However, a strong (*r* = 0.6629, *p* = 0.0367) correlation between the number of TNF+ and HHV-7+ lymphocytes has been demonstrated in the samples of the HHV-7 PCR+ group, suggesting the significance of the immune system reaction to a foreign antigen. Furthermore, the vulnerability of vascular endothelial cells, especially in the case of infection reactivation, is shown in the given study. Available serological data suggest that primary herpesvirus infection occurs early in childhood and results in a lifelong infection [24,52,53,54].

This is consistent with the results of our study. The present study demonstrated the presence of persistent HHV-7 infection in 81.5% of all enrolled OA patients. Similar results were published by Sánchez-Ponce and colleagues conducting studies on beta and gamma human herpesviruses in the case of organ transplantation, and they raised awareness about an increased risk of graft rejection [55].

Our previous studies have pointed out that the eradication of this infection often does not occur, and the virus remains in a latent state [56,57]. Complications associated with virus reactivation involve a wide range of diseases, including joint pathologies; however, the role of HHV-7 has not been completely understood thus far. Other authors have not succeeded in attempts at isolating DNA from herpesviruses in patients with OA, but have showed the presence of DNA from HSV1-2 and VZV in RA and axial spondyloarthritis patients [33]. The strength of our study includes a look at the synovial cellular constituents targeted in the case of latent or active HHV-7 infection. In our study, molecular virology data confirmed that either latent or active HHV-7 infection was coupled to immunohistochemical detection of the viral antigen in the synovial membrane. We observed that HHV-7 latency was characterized by the contribution of inflammatory cells and the presence of CD4-positive cells, establishing a strong, positive correlation with TNF-producers. Simultaneously, we proved that different cellular targets, vascular beds, and their constituents, are more vulnerable and are therefore affected in the case of active HHV-7 infection.

Previous studies have demonstrated that human herpesviruses sustain latency in cells of the hematopoietic lineage, but become reactivated upon immune challenge to cause disease [58]. Synovial macrophages are of great interest in this context since they may act as a reservoir during the period of viral infection latency. This feature has been widely explored, and evidence that even a suppressed viral transcription program exhibits several viral genes that are expressed during latent infection at the protein level has been obtained; many of these have profound effects on the cell and its environment, regulating numerous cellular functions [59,60]. Furthermore, our recent studies provided some insight into the controversial issue related to the chromosomal integration of HHV-7 [61]. In this context, the stimulation of inflammation and oxidative stress are areas of interest when suggesting the contribution of herpesviruses infections to chromosomal telomere attrition [62,63]. Previous studies have proved the role of macrophages in the development of synovial lesions and progression of OA through the production of inflammatory mediators, growth factors, and metalloproteinases, resulting in enhanced cartilage degeneration and osteophyte formation [13]. Furthermore, immunohistochemical studies have produced additional evidence of the presence of TNF, which is the key pro-inflammatory factor released by synovial macrophages, in OA [38,64]. OA synovitis has been recognized as a cytokine-driven disease, especially regarding TNF, even when manifesting with lower levels of pro-inflammatory mediators compared to that in RA [15]. A higher expression of the inflammatory mediators was found in the lining layer, and a lower expression was found in the sublining layer of the lesioned synovium [65,66]. In this study, we found a large number of TNF-labeled synovial lining cells, whereas most of the sublining constituents revealed perivascular localization. Our data are consistent with the results obtained by these authors. Furthermore, more substantial synovial macrophage infiltration demonstrated in patients with early OA, when compared to advanced OA [13], and higher levels of inflammatory cytokines, including IL-6, IL-1, and TNF, were evidenced in early compared to advanced OA [67]. The significance of findings depicting the distribution and functions of synovial macrophages and infiltrating lymphocytes has been pointed out in the given study, without denying other accurate and sensitive tools for visualizing the inflammatory cells in OA [13].

By emphasizing the significance of the immune system reaction to a foreign antigen, one may expect the appearance of system overreaction, causing potentially more harm than the viral agent itself. There is an increasing appreciation of the importance of cellular plasticity on the one hand, and the diversity of strategies and molecular mechanisms for escaping detection by the immune system evolved by HHV-7 on the other [68], suggesting the necessity for further research.

## 4. Materials and Methods

### 4.1. Patients’ Characteristics

Fifty-four patients that presented with advanced OA and underwent joint replacement surgery for the disease at the Riga East University Hospital Clinic “Gailezers” between March 2019 and January 2020 were enrolled in the study. The inclusion criterion was a primary or previous diagnosis of OA established in the given hospital. All OA subjects fulfilled relevant American College of Rheumatology (ACR) criteria for the disease-affected joints: Hip [69] and knee [70]. All subjects enrolled in the study (mean age 69 (range 35–85 years), standard deviation (SD) ± 12.28 years); 19 (35.2%) males and 35 (64.8%) females) had OA confirmed clinically and radiologically. They did not reveal any objective and subjective evidence of any other inflammatory disease apart from OA. The clinical data of patients included information on the duration, course, and clinical features of the disease at the time of presentation, whereas laboratory analyses employed to monitor OA progress included complete blood count, hemoglobin, and C reactive protein (CPR) data. PCR data were obtained from 48 out of 54 patients. These OA patients were subdivided into two study groups: HHV-7 PCR+ (*n* = 19) subjects (the first group) and HHV-7 PCR‒ (*n* = 29) subjects (the second group), accordingly. The average age of OA subjects of the first group was 68.95 years (SD ± 9.35), whereas that of the second group was 63.93 years (SD ± 12.9). The study was approved by the Ethical Committee of Riga Stradins University (Decisions No. 6-2/7/9 and No. 6-1/01/62) and was conducted according to the Declaration of Helsinki.

### 4.2. Blood Sample Collection and Detection of TNF and IL-6 Levels

Ethylenediamine tetraacetic acid anti-coagulated peripheral blood samples from OA patients were collected. Plasma samples were separated from peripheral blood by centrifugation. The levels of TNF and IL-6 (pg/mL) were measured by an enzyme-linked immunosorbent assay (ELISA) (TNF-α by Nordic Biosite, Copenhagen, Denmark), according to the manufacturer’s guidelines, and analyzed in comparison with the presence or absence of HHV-7 infection markers. The optical density was measured by a microplate reader (Multiscan Ascent, Thermo Electron Corporation, Waltham, MA, USA) at a 450 nm wavelength using Ascent Software, and the results were calculated using Microsoft Excel.

### 4.3. Nested Polymerase Chain Reaction

A nested polymerase chain reaction (nPCR) was used for the qualitative detection of the viral genomic sequence in DNA isolated from whole blood, synovial membrane tissue samples (a marker for persistent latent infection), and cell-free blood plasma (a marker of active infection). Total DNA was isolated from WPB and synovial membrane tissue samples using the standard phenol-chloroform extraction. The QIA amp DNA Blood Mini Kit (Qiagen, Hilden, Germany) was used to extract DNA from plasma. The concentration of extracted DNA was measured spectrophotometrically (Nanodrop ND-1000 Spectrophotometer, Thermo Fisher Scientific, Waltham, MA, USA). To assure the quality of the whole blood, cell-free blood plasma, and synovial tissue DNA, as well as to exclude the contamination of plasma DNA by cellular DNA debris, a β-globin PCR was carried out using a polymerase chain reaction (PCR) (C1000 Touch Thermal Cycler, BioRad, Hercules, CA, USA). One microgram of whole blood and synovial tissue DNA, as well as 10 μL of plasma DNA, were subjected to nPCR with the HHV-7-specific primer, as described previously [71]. Positive (HHV-7 genomic DNA; ABI, Columbia, MD, USA) and negative controls (DNA obtained from practically healthy HHV-7-negative donors and a reaction without template DNA), as well as water controls, were included in each experiment. In the experiments, the sensitivity of HHV-7-specific primers corresponded to one copy of HHV-7 per reaction [72].

### 4.4. Light Microscopy and Immunohistochemistry

Synovial membrane tissue specimens (*n* = 54) were obtained from all OA patients undergoing joint replacement surgery. Two series of histological sections of 4–5 μm were cut from 10% formalin-fixed, paraffin-embedded tissue samples and mounted on SuperFrost Plus slides (Germany Menzel GmbH, Braunschweig, Germany) for histopathological and immunohistochemical evaluation. Before immunostaining, deparaffinization and hydration were conducted in xylene and graded alcohol to distilled water. During hydration, a 5 min blocking process for endogenous peroxidase was conducted with 0.3% (*v/v*) H2O2 in 95% methanol. Heat-induced epitope retrieval was accomplished with the sections immersed in 10 mM sodium citrate buffer, pH 6.0, at 96–98 °C for 5 min in a vapor lock.

Immunohistochemistry was performed conventionally using a monoclonal anti-HHV-7 antibody (Advanced Biotechnologies, Columbia, MD, USA, 1:500) raised against the tegument protein pp85 of HHV-7 [73,74]; the polyclonal rabbit anti-human TNF antibody (Biorbyt, Cambridge, UK, 1:100), which labels a certain peptide of human TNF [75]; monoclonal mouse anti-human CD68 (DacoCytomation, Glostrup, Denmark, clone PG-M1, 1:50), which labels monocytes/macrophages via the recognition of lysosome proteins,; and monoclonal rabbit anti-human CD4 (Cell Marque, Rocklin, CA, USA, SP35, 1:100), which recognizes a 55 kD glycoprotein expressed on the cell surface of T-helper/regulatory T-cells.

The amplification of the primary antibody and visualization of reaction products were performed by applying the HiDef Detection HRP Polymer system and diaminobenzidine tetrahydrochloride substrate kit (Cell Marque, Rocklin, CA, USA). The sections were counterstained with Mayer’s hematoxylin, washed, mounted, and covered with coverslips. Immunohistochemical controls included the omission of the primary antibody. Sections were photographed by a Leitz DMRB bright-field microscope using a DFC 450C digital camera or scanned with a Glissando Slide Scanner (Objective Imaging Ltd., Cambridge, UK) with a 10×, 20×, and 40× objective. Reproducible measurements of tissue markers were obtained using the, Aperio ImageScope program v12.2.2.5015, Leica Biosystems Imaging, Vista, CA, USA and images were processed with the ImageJ program (National Institute of Health, Bethesda, MD, USA). Assessment of the histopathology and immunostaining was performed by two independent observers blinded to clinicopathological data.

Cells that were labeled with the anti-HHV-7, anti-TNF, anti-CD68, and anti-CD4 antibody and displayed brown reaction products were considered as immunopositive. The total number of immunopositive cells appearing within the microscopic field, depicting a certain synovial region, was estimated quantitatively in 10 randomly selected visual fields of each sample (magnification 400×).

Additionally, to better visualize the cellular distribution and localization of the HHV-7 antigen, the synovial tissue specimens were processed for fluorescent immunohistochemical staining and confocal microscopy. The sections that immunoreacted with the primary antibody overnight at 4 °C were washed in PBS, followed by incubation in goat anti-mouse IgG-FITC: sc-2010 (Santa Cruz Biotechnology, Inc., Santa Cruz, CA, USA 1:300) as the secondary antibody. Then, sections were counterstained with 4’,6-diamidino-2phenylindole (DAPI) (Thermo Fisher Scientific, Invitrogen, Renfrew, UK, 1:3,000) and mounted in Prolong Gold with DAPI (Thermo Fisher Scientific). Imaging was performed using an Eclipse Ti-E confocal microscope (Nikon, Tokyo, Japan).

### 4.5. Scoring of Synovitis by Krenn

To define synovitis, involving inflammatory changes of the synovial membrane depicting intra-articular changes of a joint, we graded it using the scoring system introduced by Krenn and Morawietz [76]. Routinely (with hematoxylin and eosin), stained slides were used, and the lesions found in the synovial membrane were assessed. The following histopathological features were evaluated and scored: The cellular hyperplasia of the lining layer; the cellular density of the sublining layer; and the presence of inflammatory infiltration: 0—absent, 1—mild, 2—moderate, and 3—strong. The sum obtained provided the synovitis score, which was interpreted as follows: 0–1, no synovitis; 2–4, low-grade synovitis; and 5–9, high-grade synovitis.

### 4.6. Statistical Data Analysis

To better interpret molecular virology, serology, histopathology, and immunohistochemistry data, statistical analyses were performed using The GraphPad Prism 8 demo version (GraphPad Software, La Jolla, CA, USA). The D’Agostino and Pearson, Anderson–Darling, and Shapiro–Wilk tests were used to evaluate whether the collected numerical data were normally distributed. If data were not normally distributed, we used nonparametric one-way ANOVA on ranks or Kruskal–Wallis test followed by the two-stage step-up method of Benjamini, Krieger, and Yekutieli as post hoc tests when comparing medians instead of means. The chi-square test was performed for categorical variables. Categorical parameters were expressed as frequencies and percentages. The results of the histopathological assessment of Krenn scores in the synovial membrane samples of the study groups are expressed as violin plots, the median, and the interquartile range (IQR) as dispersion characteristics. To compare numerical values between two groups, the nonparametric two-tailed Mann–Whitney U test was applied. In the case of paired group comparisons, the Wilcoxon matched-pairs signed rank test was used. Correlations between the numbers of immunopositive cells were determined using either parametric Pearson’s or nonparametric Spearman’s correlation analyses, depending on the data distribution. The correlations were considered as follows: 0.2 to 0.4—weak; 0.4 to 0.7—moderate; and 0.7 to 0.9—strong. A *p*-value of less than 0.05 (*p* < 0.05) was considered statistically significant.
